# In Darkest England

**Published:** 1898-12

**Authors:** 


IN DARKEST ENGLAND.—"On May 31, 1898," says Mr. Havelock Ellis,* the distinguished scholar and writer, "Mr. George Bedborough was arrested for selling to a disguised detective a copy of my book *Sexual Inversion.* He was charged before Sir John Bridge at the Bow Street Police Court with 'publishing an obscene libel' (in other words, circulating an indecent work) 'with the intention of corrupting the morals of Her Majesty's subjects.' Mr. Bedborough was simply a seller of the book, it must be added, and in no way responsible for its production.

"It was shown by the evidence of the police themselves that the sale of the book was effected in a private house, with closed doors, and that neither the book in question nor any other books were exposed for sale, or announced for sale, in the window or elsewhere. No commercial transaction could conveniently be effected with less publicity. So that if the sale of my book could be regarded as improper under such circumstances, there were practically no circumstances under which it could well be regarded as proper. Thus,

*Pamphlet. "A note on The Bedborough Trial."

although the police took no direct action against the author, publishers, and printers of the book, the effect of their action was calculated to be as fatal to the book as though they had proceeded directly against its producers.

Mr. Bedborough was committed for trial, after the magistrate had extracted from Mr. Avory, who defended, the unusual declaration that he was personally prepared to answer for the scientific character of the book, and the defence was reserved. Bail had at first been refused, although there was not the slightest reason to imagine that Mr. Bedborough, a law-abiding citizen, had any desire to put himself in the wrong by fleeing from the hands of justice; it was only accepted ultimately in the large sum of two sureties for £500; while, as is well known, dangerous criminals against the person are often easily admitted to bail in very small sums. This is a tribute to the awe which surrounds ideas, and may well be gratifying to those who live much in the world of ideas. I should add that the incriminating passages, when read out in court, proved to be simple statements of facts, mostly from the early life of the cases of inversion recorded in the volume, and my responsibility for them merely lay in the fact that I judged them to contain, in bald uncolored language, the minimum of definite physical fact required in such a book, if it is to possess any serious scientific value at all. When, however, three months later, the indictment was finaly issued, it appeared that the whole book, from the first page to the last, 'and every line in such pages,' was charged as 'wicked, lewd, impure, scandalous, and obscene.' It was solely on this ground, and not on any alleged impropriety in the method of sale, that the charge was founded.

"I may here briefly state the general character of the book. It is the more necessary to do so since no undue publicity has been sought, and the book was so little known before these proceedings were taken, except to specialists, that the majority of my own friends had never heard of it until they saw it proclaimed as 'obscene' in the police news of every London newspaper.

"*Sexual Inversion,* published at the end of the year 1897, is the first volume of a series of *Studies in the Psychology of Sex,* which I projected over twenty years back, and which I have ever since had before my mind, as the serious and vitally important subject to which the best energies of my life should be devoted. The work will extend to five or six volumes, and although this first volume discusses a form of perverted sexuality, the *Studies* as a whole will deal mainly with the normal sex impulse. It should be needless to point out the magnitude and the importance of the problems arising in such an investigation; in this first volume, moreover, we are brought face to face with a practical question which is constantly demanding attention, both in society and the law courts. Whatever diffidence one may feel in approaching questions of this nature, there should be no doubt as to the necessity of so doing provided we approach them seriously.*

"How seriously I approached this great subject may be judged, not only from the long period of labor and preparation spent on the work, but from the fact that I occupied several years in the merely preliminary task of attempting to clear the ground by inquiring into the psychological and anthropological secondary sexual differences of the sexes, the main results of this special inquiry appearing in 1894 under the title of *Man and Woman.* Before its publication in England, *Sexual Inversion* had been translated into German by Dr. Kurella, a physician and criminal anthropologist of distinguished reputation, and published at Leipzig. In its final English shape it expresses my most mature convictions on the subject it treats; the opinion of judicious friends had been obtained at doubtful points, and every sentence carefully weighed. Errors of fact or opinion may possibly be found, but there is not a word which on moral grounds I feel any reason to regret or withdraw. Any question of retraction or apology could not, therefore, possibly arise; it would be a kind of intellectual suicide.

"It has been supposed by many who have never seen the book that I have attempted to popularize the study of sexual questions, and to make widely known the results obtained by other investigators. That is altogether a mistake. The book is founded on original data,

---

*Mr. Stead (*Review of Reviews*, August, 1898), in commenting on this "sudden extension of police censorship to the realm of scientific discussion," has forcibly stated the practical importance for the community of the study of such questions: "It may be alleged that such questions should not be discussed, and that the whole question should be buried in impenetrable silence. The answer to this is if the legislator makes one theory of the Psychology of Sex the basis for passing a law which sends citizens to penal servitude, it is impossible to shut out such a theory from public discussion. Dr. Ellis' inquiry goes to the very root of the theory upon which one section of the Criminal Law Amendment Act is based, and if the conclusions at which he arrives are sound the principle of that legislation is unsound, and will have to be modified, for the same reason that capital punishment is never enforced upon persons of disordered minds." This reasonable and temperate "protest" is the more note-worthy since, as is well known, it was Mr. Stead who chiefly called public attention to the necessity of passing the Act here referred to. I should add, however, that the only clause of that Act which I discuss and criticise was not part of the original Bill, but was introduced, as an amendment, on the motion of Mr. Labouchere.

and contains the first collection of cases of sexual inversion, unconnected with the prison or the asylum, which has ever been obtained in England; it is written in bald and technical language, published at a high price; and having been announced and sent for review only in special medical and scientific quarters, its existence was practically unknown to the general reader until these proceedings were initiated. There may well be, I know, a question as to the value of cloistered virtues, as to the worthiness of that innocence which is merely ignorance and vanishes at a breath, as to the rights of every adult person to full knowledge of the sexual facts of life. But that question is not raised by my work. I appealed only to doctors, to psychologists, to those concerned with medico-legal matters, and to the handful of thinkers who are interested in the social bearings of the physical and psychic problems of life. By such my work has been accepted—so far as I know at present without exception—in the serious spirit in which it was put forward. Every alienist of distinction whose opinion I have obtained has assured me of his belief in the importance of the subject, and of his sense of the scientific tone and temper in which I have dealt with it. Every medical journal in half a dozen countries which has received the book has without exception judged it favorably, and not one has suggested that I have been guilty of the slightest impropriety. I may indeed say that the medical support I have received has often been rather on moral than on scientific grounds; it has repeatedly been remarked that an English tone of reticence distinguishes this book from the other works on the same subject by continental writers. The numerous letters of gratitude for the work, and strong support of its objects, which have reached me from thinkers and social reformers, men and women, I refrain from more than mentioning; they have sufficed to show me that the aim and nature of my task are appreciated by the small class of people whom, in addition to medical readers, I have alone sought to address.

"The trial took place on the 31st of October, at the Old Bailey. An influential committee had been formed to enable the matter to be fought out as a test case involving a great principle, and a large sum of money had been subscribed for this purpose.

"Shortly before the trial, however, Mr. Bedborough, without consulting those whose support he had accepted, resolved to throw over the question of principle and, in order to obtain the best terms for himself, plead guilty to a part of the charge. Further, in consequence of other circumstances which will shortly be investigated, the defendant's counsel who from the outset had been prepared to justify the scientific character of the book, was not able to appear.

"The publisher and myself were duly represented by counsel, but having no standing in the case he was necessarily unable to speak. Thus although my book was the real subject of the trial there was no legal opportunity for any voice to be heard on its behalf.

"The defendant having pleaded guilty was released on his own recognisances. The judge, Sir Charles Hall (the Recorder) made the following remarks which I quote *verbatim:* 'You might at the outset perhaps have been gulled into the belief that somebody might say that this was a scientific book. But it is impossible for anybody with a head on his shoulders to open the book without seeing that it is a pretence and a sham, and that it is merely entered into for the purpose of selling this filthy publication.'

"I could scarcely desire stronger evidence than Sir Charles Hall has here been good enough to afford of the necessity for a resolution which I had made long before the trial took place.

"When proceedings were first taken against Mr. Bedborough I at once, with the consent of the publishers, suspended the sale of the book. Subsequently I decided, whatever the verdict might be, to continue to withhold the book for a considerable period, having no wish to avail myself of the enormous advertisement spontaneously offered by the police. Moreover, I have now decided not to publish the remaining volumes of my *Studies* in England. I propose here to state the reasons for a course which many of my friends regard as a confession of defeat.

"Intelligent spectators of life have declared that this prosecution of a book-seller for selling a purely scientific work will mark an epoch so far as our country is concerned. It has acted as a *reductio ad absurdum,* they say; it has quickened the public conscience to a finer sense of what is fitting in these matters. Henceforth public opinion will be strong enough to check at the outset any foolish interference of the police with scientific discussion. Just as a police charge of 'blasphemy,' which twenty years ago was a real serious charge, would to-day only arouse a smile, so, it is said, never again could a scientific book, issued and sold as this was, be dragged into the mire of the courts as 'obscene,' or a reputable citizen who sold such a book be haled before the magistrate on a charge of 'corrupting the morals' of his fellow subjects.

"It may be so. I would gladly believe that any action of mine had assisted my countrymen to win that intellectual freedom which is already possessed by every other civilized country except Russia. But no one can give any guarantee that such will be the fact, and life is too short to enable me to wait another twenty years to verify the prophecy.

"It must be remembered that so far as an author is concerned the injury done by such a prosecution is done in the act of bringing it. The manifold chances that befall a book on any highly specialised and technical subject, when submitted to a judge and jury, may or may not lead to the justification of the author. The injury is already done. The anxiety and uncertainty produced by so infamous a charge on a man and on those who belong to him, the risk of loss of friends, the pecuniary damages, the proclamation to the world at large, which has never known and will never know the grounds on which the accusation is made, that an author is to be classed with the purveyors of literary garbage—this power is put into the hands of any meddlesome member of that sad class against which the gods themselves are powerless.

"This is a risk to others, and a domination over myself, which I at all events have no intention of submitting to. In this country it is a sufficiently hard task for any student to deal with the problems of sex, even under the most favorable circumstances. He already, as it were, carries his life in his hands. He has entered a field which is largely given over to faddists and fanatics, to ill-regulated minds of every sort. He must, at the same time, be prepared to find that the would-be sagacity of imbeciles counts him the victim of any perversion he may investigate. Even from well-balanced and rational persons he must at first meet with a certain amount of distrust and opposition. To encounter this inevitable and legitimate opposition, and to preserve his serenity and equipose, is itself a sufficient strain on any man. It would be foolish to place oneself as well beneath the censure of an ignorant and too zealous police official, and to accept the chain of uncertain evils, and the certain public stigma, which a prosecution necessarily involves.

"Under these circumstances, therefore, the difficulties of publishing the remaining volumes of my *Studies in the Psychology of Sex* in England are sufficiently obvious, and the decision I have been forced to reach seems inevitable. To wrestle in the public arena for freedom of speech is a noble task which may worthily be undertaken by any man who can devote to it the best energies of his life. It is not, however, a task which I have ever contemplated. I am a student, and my path has long been marked out. I may be forced to pursue it under unfavorable conditions, but I do not intend that any consideration shall induce me to swerve from it, nor do I intend to injure my work or distort my vision of life by entering upon any struggle. The pursuit of the martyr's crown is not favorable to the critical and dispassionate investigation of complicated problems. A student af nature, of men, of books, may dispense with wealth or position; he cannot dispense with quietness and serenity. I insist on doing my own work in my own way, and cannot accept conditions which make this work virtually impossible. Certainly I regret that my own country should be almost alone in refusing to me the conditions of reasonable intellectual freedom. I regret it the more since I deal with the facts of English life, and prefer to address English people. But I must leave to others the task of obtaining the reasonable freedom that I am unable to attain."

---

This looks like persecution; still the same old systematic throttling of scientific investigation by bigotry and ignorance, when it is recalled that Editor Stead a few years ago ventilated, through the secular press *(Pall Mall Gazette)*, the foulest scandal relating to this very subject,—but in that case no pretense was made that it was in the interest of science,—and the secular press also pretty thoroughly ventilated the Oscar Wilde nastiness. We heard of no arrest except that of Wilde.

Yet this outrage is hardly to be wondered at, after all, when we recollect that only a short while ago Mr. Gladstone, the ablest Parliamentary leader of the century,—so enlightened in all else, still in the bondage of bigotry and superstition,—in an elaborate article attempted to controvert, by citations from Scripture, the fact long since and thoroughly demonstrated by irrefragible geological proofs that man existed on earth long ages prior to the dates assigned in Genesis to Adam.

Let Mr. Ellis come to America, the land of the free, to get his books published. It will have a tremendous sale.

We wonder if the police suppressed Krafft-Ebing's beastly book?

---

DR. WILSON'S PAPER ON TYPHOID FEVER, published in this issue, was read at the annual meeting of the State Medical Association, and like all papers presented, it was referred to the publishing committee. The paper was thrown out—rejected—not included in the yearly volume of "Transactions." Dr. Wilson naturally feels a little indignant, and is surprised at the treatment of his paper. It is worth while, perhaps, to say that the Secretary of the Association, an ex-Professor of Practice of Medicine, is chairman of the publishing committee. He has usually two associates on the committee, but most frequently they are only nominally members of the committee; the entire work of examining papers, selecting those for publication, editing them, reading the proof, etc., is left to the chairman.

The treatment accorded Dr. Wilson's paper is not calculated to encourage physicians to prepare and present papers. Dr. Wilson is an old member of the Association, an old practitioner who has had good opportunities for practical observation at the bedside. He represents a large class—unquestionably the majority of the members of the Association. He is a plain, unpretentious country practitioner, and makes no pretentions to knowing all that the books say; though he is a man of good education and extended observation.

If I understand the object in these annual comings together of the members of the Association, it is to exchange views and experiences; to discuss diseases usually met in Texas. Dr. Wilson came forward—the first time since he has been a member—and modestly gave a summary of his observation on typhoid fever in a long practice, and his conclusion. He presented his method of treating the disease, based upon those conclusions. He naturally expected to hear his paper discussed with a courtesy due to any member; doubtless hoped to learn something from the experience of brother country doctors, or perhaps to be taught by his city colleagues. That his paper was ridiculed and rejected is no credit to the Association. We are pained that any member—be he the humblest, the most unlearned, the youngest, the most inexperienced one in the lot, should be treated with such scant courtesy. Dr. Wilson is not of that class; but a safe, painstaking and conscientious physician, who has been many years in the practice. True, his knowledge of the bacteriology of typhoid fever may not be up to Osler's latest book, but in the main he is correct; the fever is a germ disease—the specific micro-organism causing it, the *bacillus typhoides* (bacillus of Eberth) being the well known and generally recognized agent. Germicidal treatment—a germicide that will kill this *bacillus* and not kill the patient, has been the dream and ultimatum of all therapeutic investigation of the subject for twenty years. Dr. Wilson suggests nitrate of ammonia, and assures us that for eight years he has used it with success. It is certainly worth trying.

We publish the paper with much satisfaction, and will be pleased to hear from other country doctors.

